# Noninvasive stimulation of the ventromedial prefrontal cortex modulates rationality of human decision-making

**DOI:** 10.1038/s41598-022-24526-6

**Published:** 2022-11-23

**Authors:** Thomas Kroker, Miroslaw Wyczesany, Maimu Alissa Rehbein, Kati Roesmann, Ida Wessing, Markus Junghöfer

**Affiliations:** 1 Institute for Biomagnetism and Biosignalanalysis, University of Muenster, Malmedyweg 15, 48149 Muenster, Germany; 2 Otto Creutzfeldt Center for Cognitive and Behavioral Neuroscience, University of Muenster, Muenster, Germany; 3 Institute of Psychology, Jagiellonian University, Krakow, Poland; 4 Institute for Clinical Psychology and Psychotherapy, University of Siegen, Siegen, Germany; 5 Department of Child and Adolescent Psychiatry, University Hospital Muenster, Muenster, Germany

**Keywords:** Cognitive neuroscience, Decision, Human behaviour

## Abstract

The framing-effect is a bias that affects decision-making depending on whether the available options are presented with positive or negative connotations. Even when the outcome of two choices is equivalent, people have a strong tendency to avoid the negatively framed option. The ventromedial prefrontal cortex (vmPFC) is crucial for rational decision-making, and dysfunctions in this region have been linked to cognitive biases, impulsive behavior and gambling addiction. Using a financial decision-making task in combination with magnetoencephalographic neuroimaging, we show that excitatory compared to inhibitory non-invasive transcranial direct current stimulation (tDCS) of the vmPFC reduces framing-effects while improving the assessment of loss-probabilities, ultimately leading to increased overall gains. Behavioral and neural data consistently suggest that this improvement in rational decision-making is predominately due to an attenuation of biases towards negative affect (loss-aversion and risk-aversion). These findings recommend further research towards clinical applications of vmPFC-tDCS as in addictive disorders.

We humans like to believe that our decision-making behavior follows rational considerations. We think that as rational people we can conscientiously analyze the consequences of available options by weighing their probabilities and comparing the results to finally choose the optimal option that maximizes our gain and minimizes our loss. However, in pioneering studies leading to the development of the ‘prospect theory’, Kahneman and Tversky showed that humans often tend to rely on heuristics and other cognitive shortcuts, instead of employing cognitively demanding evaluations^1,2^. A remarkable deviation from the behavior of an ideal rational agent (‘homo economicus’) is the so-called framing-effect, where presenting either positive or negative connotations of an option can bias decisions: For instance, when people are asked to either opt for a treatment A which would *save* 400 of overall 600 infected patients or for treatment B with which 200 of 600 patients would *die*, a vast majority would prefer treatment A, which is positively framed, but still identical to B in terms of outcomes^3^. One possible mechanism underlying the framing-effect can be loss-aversion as humans typically rate losses as more salient as gains of the same amount^1,2,4^. This cognitive bias of loss-aversion likely roots in an asymmetric evolutionary pressure on losses and gains: “when survival is uncertain, marginal losses prove more critical for reproductive success than marginal gains”^5^. Further biases modulating the framing-effect are the so-called risk-aversion bias for gains and the risk-seeking bias for losses^1,2,4^. Because the increase of the subjective utility compared to the objective value decreases with increasing values (e.g. the difference of subjective utility between gains of 50 and 55€ is smaller compared to the difference between gains of 10 and 5€), humans tend to prefer smaller wins over gambling for a greater amount, although the expected value of the gambling option would be higher (i.e. humans typically show risk-aversion for gains). As the same relation holds for large compared to small losses, people typically tend to prefer to gamble for greater amounts than accept smaller losses although the expected loss of the safe option would be lower (i.e. humans show a risk-seeking bias for losses).

The impact of the framing-effect and reactions to odds—and therefore the modulation of rational decision-making by any kind of intervention—can nicely be investigated in gambling studies that can consider gains and losses simultaneously^3,6^. For example, if participants have to decide between ‘*lose* 60ct of 100ct’ or ‘gamble for 100ct with 20% probability’ they would typically avoid the negatively (‘*lose’*) framed option and gamble for the whole amount, despite a higher expected safe residual gain (40 cents) in contrast to the expected gain (20 cents) of the gambling decision. Inversely, most participants would choose the positively framed ‘*keep* 40ct of 100ct’ instead of ‘gamble for a 100ct with 80% probability’, despite the expected gain of the gambling option (80 cents) would be higher than the safe gain of 40 cents.

Studies investigating the underlying neural correlates of the framing-effect, loss-aversion and risk-aversion/risk-seeking in gambling studies consistently identified the ventromedial prefrontal cortex (vmPFC) as a cardinal player. For example, vmPFC activity was greater in individuals with greater risk-aversion^7^ and neural activity in the vmPFC decreased with increasing potential losses and the strength of this association predicted individual loss-aversion^8^. Consistently, individuals with increasing susceptibility to the framing-effect showed decreased activity in the vmPFC^6^. The finding that patients with vmPFC lesions feature an increased framing-effect relative to patients with other lesions and healthy controls^9^ adds causality to this correlational evidence.

Another aspect of rational decision-making that is dependent on the vmPFC is the inhibition of impulsive and short-sighted choices. Patients with vmPFC lesions for instance show a distinct insensitivity to consequences of decision-making and are primarily guided by immediate prospects^10^. These patients also show increased risk-taking and bet more money in gambling studies than healthy controls^11^. This causal link between vmPFC dysfunctionality and irrational economic behavior is further supported by the finding that the reduction of rational economic decision-making with aging correlates with gray matter volume reduction in ventral PFC regions^12^.

Since reduced vmPFC activity and vmPFC dysfunctions have been associated with irrational decision-making, as indexed by an increased susceptibility to framing-effects and poorer assessments of the odds, it appears tempting to assume that excitatory tDCS over the vmPFC (i.e. increasing vmPFC excitability) might have mitigating effects on these biases and might improve rational decision-making. Because the vmPFC is an almost unique structure in coding gains with activity increase and losses with activity decrease^8,13^, the vmPFC recommends itself as ideal target for valence specific interventions via brain stimulation (i.e. modulation of valence biases such as framing). In fact, a series of fMRI and MEG studies from our lab recently revealed that excitatory compared to inhibitory stimulation of the vmPFC attenuated behavioral and neural negativity-biases to emotional scenes and emotional facial expressions in healthy participants^14–17^.

Here we combined the financial gambling paradigm developed by Kahneman and Tversky^10^—and adopted by DeMartino and coworkers^6^ as well as Pujara and colleagues^9^—with the vmPFC-targeted stimulation and MEG neuroimaging approach as used in our previous studies^14–17^. Within this paradigm, participants decided to either accept a safe amount of an initial stake or gamble for the whole amount with variable risks to win or lose. The safe option was either framed in a positive or negative way with identical net gains for both frames. The probability of taken risks thus informed about the susceptibility to the framing-effect and the consideration of odds which here were operationalized as indices of rational decision-making. Participants finally received feedback about gains and losses. The participants’ evaluation of gains and losses informed about the strength of the framing-effect in this feedback phase which was used as further index of rational decision-making.

Directly preceding the financial gambling task, one half of the participants received excitatory vmPFC-targeted stimulation at a first session and inhibitory tDCS over the vmPFC at a second session while the other half received the reversed order. We hypothesized that vmPFC excitation compared to vmPFC inhibition by transcranial Direct Current Stimulation (tDCS) would result in a relatively attenuated framing-effect (i.e. predominately a decreased tendency to gamble in the ‘loss-frame’ and potentially an increased tendency to gamble in the ‘gain-frame’ and thus overall more rational decision-making) as well as an improved consideration of odds and ultimately higher gains.

Investigations of event related potentials (ERPs) of gain and loss processing during gambling tasks have consistently identified ERP components at mid-latency to late time intervals (around 250 ms after feedback) and at central to frontal-central scalp regions^18–20^. While these components typically reflect responses to both positive and negative feedback, stronger reactions to losses as compared to gains, most presumably reflecting loss-aversion, have been identified in various ERP studies^18,19^. Thus, MEG correlates of attenuated framing after excitatory stimulation were expected to occur at prefrontal regions and mid-latency to late time intervals. Since previous research has shown that the vmPFC mediates rationality by inhibiting maladaptive responses (i.e. irrational loss-aversion or choosing high-risk options^21,22^; we expected that the neural data would reflect an enhanced ability of prefrontal brain regions to inhibit framing-effects (in the decision-phase and feedback-phase) and thus maladaptive choices (in the decision-phase) following excitatory compared to inhibitory stimulation.

With the focus on effects of vmPFC-targeted stimulation on rational decision-making and feedback-processing, behavioral and neural effects which were modulated by the stimulation are reported in the following results section. For main effects and further interactions please consult the supplementary material (SM).

## Results

### Non-invasive vmPFC-tDCS modulates rational decision-making

**Figure 1:**
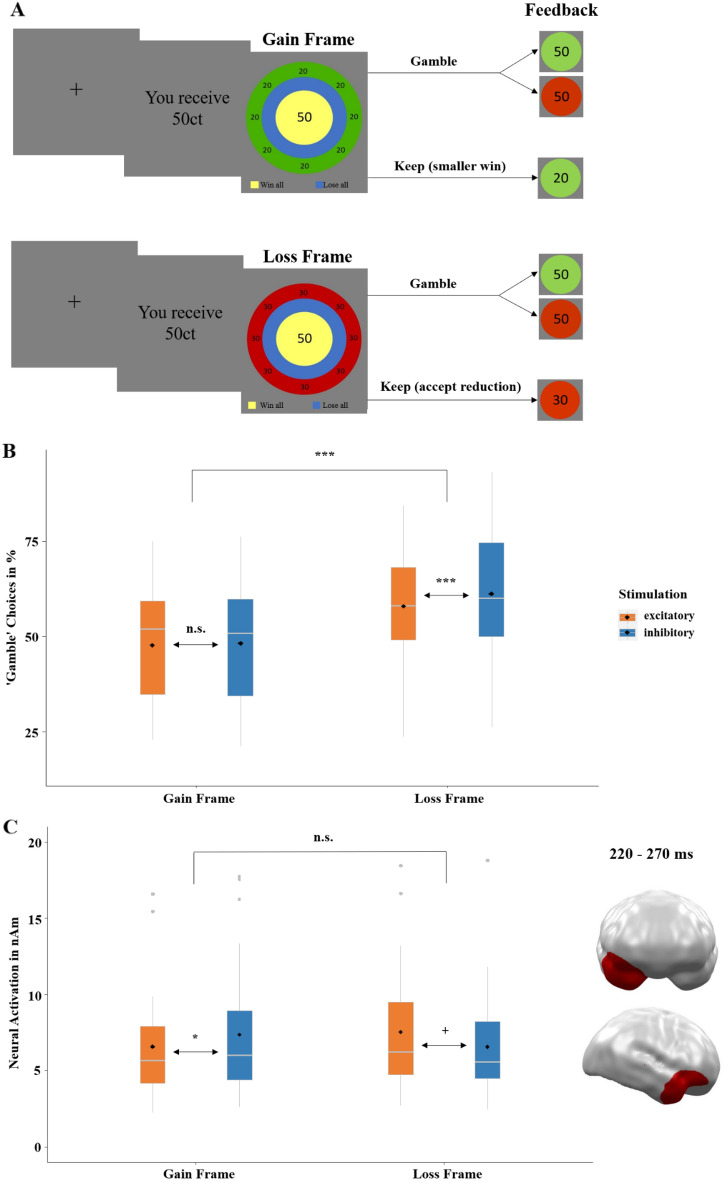
(**A**) Course of a single trial in the gambling task adapted from DeMartino and colleagues^6^. Each trial began with a fixation cross presented for 500 ms, followed by the presentation of the ‘game stake’ of 25, 50, 75 or 100 cents. The subsequent ‘choice stimulus’ reminded participants of the initial amount (center), the chance to win or the risk of losing when choosing the ‘gamble’ option (20, 40, 60, 80%, based on the relative sizes of the blue and yellow circles), and the frame when choosing the safe ‘keep’ option (green and red outer ring for gain and loss-frames, respectively). After choosing the ‘keep’ or ‘gamble’ option, feedback on the win or loss was given via green (win) and red (loss) circles, with the amount depicted in the center. Stimuli were placed centrally to minimize eye movements and related MEG artifacts. MEG correlates of neural activity evoked by the choice and the feedback stimuli were analyzed. (**B**) Proportion of ‘gamble’ choices for gain- and loss-framed trials in percentage. An ideal rational agent would have chosen the gain-frame option and the loss-frame option in equal frequency, as both resulted in identical wins or losses. However, replicating a strong deviation from rationality, participants chose the risky ‘gamble’ option in the loss-framed condition much more often. Importantly, this framing-effect (i.e., a preference for gambling in the loss-frame compared to in the gain-frame) was stronger after inhibitory than excitatory stimulation of the vmPFC. Convergent with the idea that vmPFC-targeted stimulation modulates loss-aversion, the effect of excitation compared to inhibition of the vmPFC was highly significant in the loss-frame but not in the gain-frame. (**C**) Significant spatio-temporal cluster in right anterior temporal/orbitofrontal areas featuring an interaction effect of stimulation by frame. The relatively greater neural activation in response to the loss-frame after excitatory compared to inhibitory tDCS suggests that vmPFC excitation results in more elaborate inhibition of the loss-frame processing than in vmPFC inhibition, such that excitation leads to a reduced saliency of the loss condition and, eventually, a reduced framing-effect, as seen in (**B**). The location of this cluster agrees well with results of the DeMartino and coworkers^6^, in which enhanced activity in the vmPFC as well as in right OFC regions was associated with more rational decision-making. Topographies of effects observed in L2-MNE were projected on standard 3D brain models for visualization. Boxplots indicate means (black dot), medians (grey line) and lower and upper quartiles. Asterisks indicate significance levels: +  < 0.1, * < 0.05, ** < 0.01, *** < 0.001.

We applied a within-subjects design so that each participant received excitatory and inhibitory stimulation of the vmPFC on two different days with at least 48 h in between (Fig. 4). The tDCS was optimized for targeting the vmPFC and minimizing the impact on other brain regions (Fig. 5). In both excitatory and inhibitory conditions participants were stimulated with a current strength of 1.5 mA over 10 min and were unable to differentiate between stimulation conditions (see SM2.1). Immediately after stimulation, participants performed the gambling task, where gambling behavior and event-related magnetic fields were measured. At the beginning of each trial, participants received an initial amount (‘gambling stake’), which was varied (25ct, 50ct, 75ct, 100ct) to enhance the credibility of the gambling paradigm (Fig. SM2). Participants were asked to either keep a smaller amount or to gamble for the full amount. The 'keep' option was either framed as a gain (Fig. 1A; gain-frame: receive a smaller but safe amount) or framed as a loss (Fig. 1A; loss-frame: subtraction of a smaller safe amount). Although the final monetary amounts were equivalent in both frames, participants chose the ‘gamble’ option in the loss-frame and accepted the safe smaller amount in the gain-frame more often, replicating the framing-effect^10^ (Fig. 1B; *z* = 2.64 , *p* = 0.008). Next, we specifically tested the hypothesis that noninvasive vmPFC-targeted stimulation modulates the framing-effect as index of rational decision-making. Indeed, a logistic regression with the predictors of stimulation (excitatory, inhibitory) and frame (gain-frame, loss-frame) revealed a significant interaction (*z* = 2.19, *p* = 0.029) modulating the decision (keep, gamble). Importantly and supporting our hypothesis, excitatory vmPFC-targeted stimulation resulted in a significantly reduced framing-effect compared to inhibitory stimulation (Fig. 1B; *χ*^2^ = 14.74, *p* < 0.001). Furthermore, excitatory compared to inhibitory stimulation significantly reduced the proportion of gambling choices in the loss-frame (*χ*^2^ = 12.24, *p* < 0.001) but not in the gain-frame (*χ*^2^ = 0.086, *p* = 0.769) supporting the hypothesis that reduced salience of negative stimuli after excitatory stimulation underlies the attenuated framing-effect.

To uncover underlying neural correlates of the framing-effect and its interaction with stimulation, we computed a 2 × 2 × 2 repeated-measures ANOVA on source-localized MEG data (the dependent variable; see SM1.4) with the factors stimulation (excitatory, inhibitory), frame (gain-frame, loss-frame) and decision (keep, gamble). The interaction of stimulation by frame was significant at the junction of right anterior temporal and right orbitofrontal regions in a mid-latency time interval between 220 and 270 ms (*p*-cluster = 0.023; Fig. 1C). Post-hoc t-tests of this interaction revealed less activation in the gain-frame after excitatory compared to after inhibitory stimulation (*t* = 1.77, *p* = 0.044), while activation in the loss-frame showed the opposite trend-significant effect (*t* = − 1.65, *p* = 0.055). The relatively greater neural response to the ‘loss-frame’ after excitatory tDCS suggests a potentially improved inhibition of loss-aversion, most probably leading to a reduced salience of losses and eventually resulting in an attenuated framing-effect as seen in the behavioral stimulation effects. The location of the cluster nicely converges with fMRI findings of DeMartino and coworkers^6^, who showed that, in addition to vmPFC areas, enhanced activity at right orbitofrontal cortex regions (OFC) was also associated with more rational decision-making as reflected by reduced framing-effects. In short, these findings suggest that a modulation of vmPFC excitability influences reactions to framing on the behavioral level in the form of changed decision-making and on the neural level at right OFC regions.

**Figure 2:**
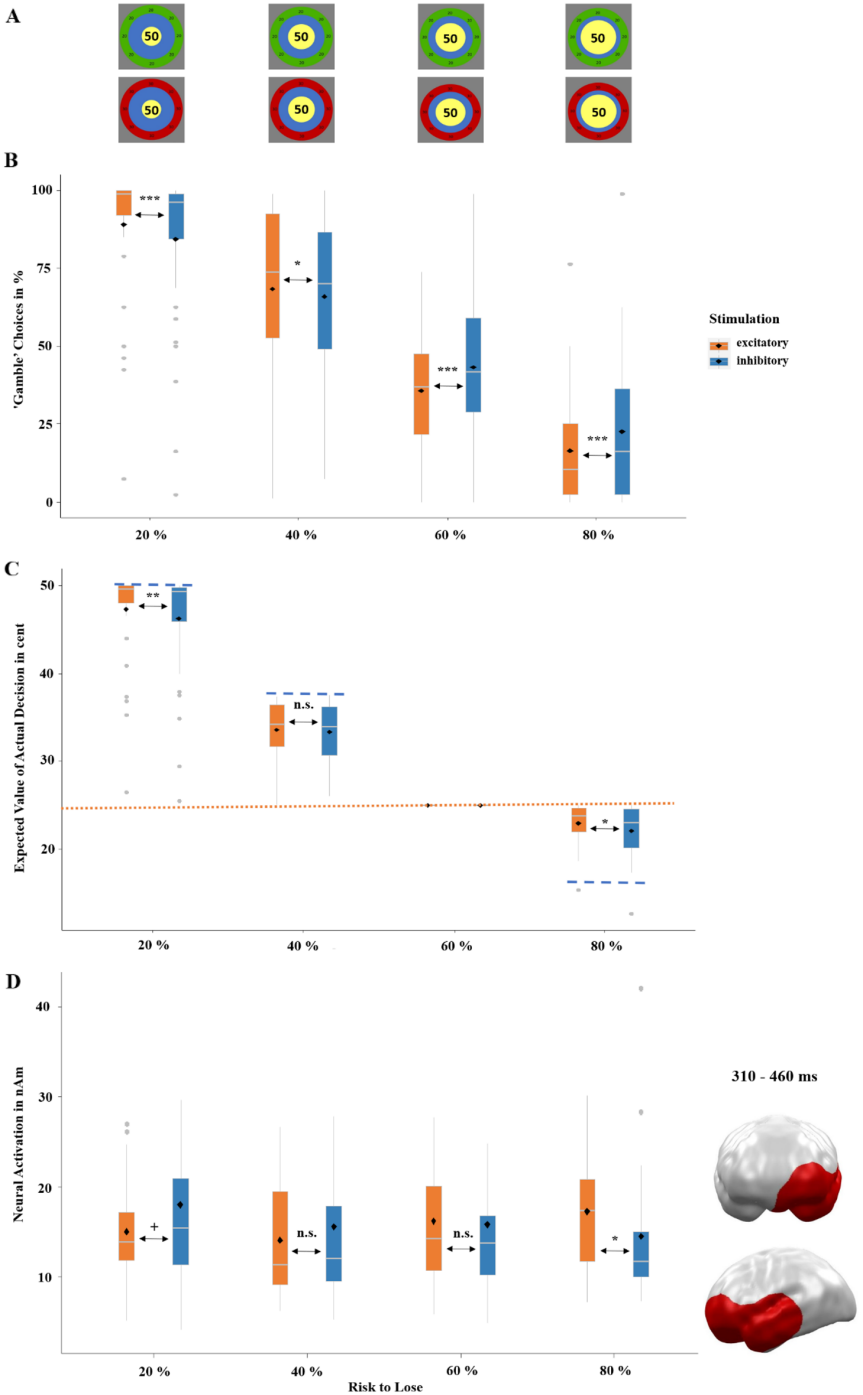
(**A**) The relative risk-to-lose percentages or chance-to-win percentages, respectively, when choosing the ‘gamble’ option was based on the relative sizes of the blue and yellow inner circles of the choice stimulus. (**B**) Proportion of ‘gamble’ choices in percentage depending on the respective risk-to-lose. After excitatory stimulation, participants gambled more often at the lower risk-to-lose conditions (20 and 40%; i.e. risk-aversion bias for rather expected gains was reduced) but avoided gambling more often at the higher risk-to-lose conditions (60 and 80%; i.e. loss-aversion bias for rather expected losses was reduced). (**C**) Mean expected values of actual choices in cents depending on the respective risk-to-lose conditions. The mean expected outcome for the ‘keep’ option averaged across all initial amounts was 25 cents (dotted orange line in **C**). The mean expected outcome of the ‘gamble’ option across all initial amounts was 50 cents at 20% risk, 37.5 cents at 40% risk, 25 cents at 60% risk and 12.5 cents at 80% risk (dashed blue lines in **C**). Concluding, to maximize the expected value (i.e., their overall winnings) participants should have always chosen the ‘gamble’ option in the 20 and 40% risk conditions and should have always chosen the ‘keep’ option in the 80% risk condition (in the 60% risk condition, the ‘keep’ and ‘gamble’ options led to identical averaged wins). Without stimulation, participants performed already quite rationally, as they almost reached the maximal wins for each risk-to-lose condition. However, in the low-risk 20% condition and the high-risk 80% condition, participants reached significantly higher winnings after excitatory compared to inhibitory stimulation. As the ‘keep’ and ‘gamble’ choices both resulted in 25 cents for the 60% condition, the respective expected values were always identical and are shown for clarity reasons only. (**D**) Significant spatio-temporal cluster at left prefrontal and anterior temporal areas featuring an interaction effect of stimulation by risk-to-lose condition. Integration of these neural responses with the behavioral results (**B** and **C**) suggests that excitatory (versus inhibitory) stimulation gave participants a greater ability to inhibit inadequate risky behavior in the high-risk 80% condition, while it reduced the inhibition of risky behavior (i.e., risky behavior was facilitated) in the low-risk 20% and semi-low-risk 40% conditions. Thus, convergent with the behavioral effects, this neural pattern suggests that excitatory compared to inhibitory stimulation relatively reduced risk-aversion for rather expected gains and reduced risk-seeking for rather expected losses together facilitating rational decision-making toward the expected value (**C**). Topographies of effects observed in L2-MNE were projected on standard 3D brain models for visualization. Boxplots indicate means (black dots), medians (grey lines) and lower and upper quartiles. Asterisks indicate significance levels: +  < 0.1, * < 0.05, ** < 0.01, *** < 0.001.

Next, we aimed to investigate whether the more rational decision-making following excitatory versus inhibitory vmPFC-targeted stimulation as shown above in the ‘keep’ option generalized to decision-making depending on the odds (i.e. when choosing the ‘gamble’ option). Here, the risk-to-lose or the chance-to-win respectively was varied in steps of 20, 40, 60 and 80%, indicated by the size relation of the inner blue and yellow circles in both frames (Fig. 2A). Of course, participants increasingly avoided gambling with increasing risk-to-lose (*z* = − 9.60, *p* < 0.001). But importantly, the effect of risk-to-lose on gambling behavior was significantly modulated by stimulation (risk-to-lose by stimulation: *z* = 6.60, *p* < 0.001; Fig. 2B). Post-hoc tests revealed more risky-choices in the two low-risk conditions (20%: *χ*^2^ = 35.81, *p* < 0.001 and 40%: *χ*^2^ = 4.81, *p* = 0.028) but fewer risky-choices in the two high-risk conditions (60%: *χ*^2^ = 37.32, *p* < 0.001 and 80%: *χ*^2^ = 40.97, *p* < 0.001). Thus, excitatory compared to inhibitory stimulation reduced the loss-aversion and/or risk-seeking bias for rather expected losses (i.e. high- and medium risk to lose trials) and reduced the typical risk-aversion bias for rather expected gains (i.e. high- and medium chance to win trials).

A representation of the averaged winnings achieved in the game across all choices (‘keep’ or ‘gamble’; Fig. 2C) elucidates the consequences of the participant’s behavior. A constant choice of the ‘keep’ option would have resulted in averaged winnings of 25 cents across all risk conditions (Fig. 2C dotted line). A constant choice of the ‘gamble’ option would have resulted in averaged winnings of 50 cents at 20% risk, 37.5 cents at 40% risk, 25 cents at 60% risk and 12.5 cents at 80% risk (Fig. 2C dashed lines). Thus, participants behaved quite rational as they almost reached the maximal win for all risk conditions. However and importantly, a t-test across all trials (*t*(11,519) = 2.23; *p* = 0.026) revealed that after excitatory vs. inhibitory stimulation averaged wins were significantly higher. Post-hoc t-tests showed significantly higher winnings in the lowest 20% (*t* = 1.74, *p* = 0.041) and predominately in the highest 80% (*t* = 2.95, *p* = 0.002) risk conditions after excitatory compared to inhibitory stimulation. Averaged winnings did not differ in the 40% condition (*t* = 0.62, *p* = 0.267) and could not differ in the 60% risk-to-lose conditions because both ‘keep’ and ‘gamble’ choices resulted in an identical amount of 25ct. In short, greater winnings after excitatory compared to inhibitory stimulation seem to result from more accurate risk assessment in both loss and gain frames. The behavioral results remain qualitatively equivalent in a full model including the predictor initial amount, as indicated in SM2.3.1.

On the neural level, we performed a 2 × 4 repeated-measures ANOVA with the factors stimulation (excitatory, inhibitory) and risk-to-lose (20, 40, 60, 80%) on source-localized MEG data (dependent variable). This analysis revealed an interaction of stimulation by risk-to-lose between 310 and 460 ms (Fig. 2D) at left anterior temporal and left orbitofrontal regions (*p*-cluster = 0.025) and thus later but laterally symmetric to the above reported interaction of stimulation by frame (Fig. 1C). Post-hoc tests of neural activity within this cluster revealed that this interaction was driven by the lowest and highest risk conditions (20%: *t* = − 1.56, *p* = 0.065; 80%: *t* = 1.85, *p* = 0.038), while the medium risks, did not show significant effects of stimulation (40%: *t* = − 0.82, *p* = 0.209; 60%: *t* = 0.23, *p* = 0.409). Convergent with the behavioral effects, this neural pattern suggests that excitatory compared to inhibitory stimulation facilitates rational decision-making towards maximized winnings.

Taken together, the behavioral data consistently indicates a modulation of decision-making towards increased rationality after excitatory compared to after inhibitory vmPFC-targeted stimulation. The reduced framing-effect and greater ability to estimate risks is mirrored by the neural data providing relatively enhanced inhibition of reactivity to negative stimuli (i.e. potentially irrational loss-aversion) and high-risk options after excitatory versus inhibitory stimulation.

### Non-invasive vmPFC-tDCS modulates the framing-effect in feedback-processing

**Figure 3:**
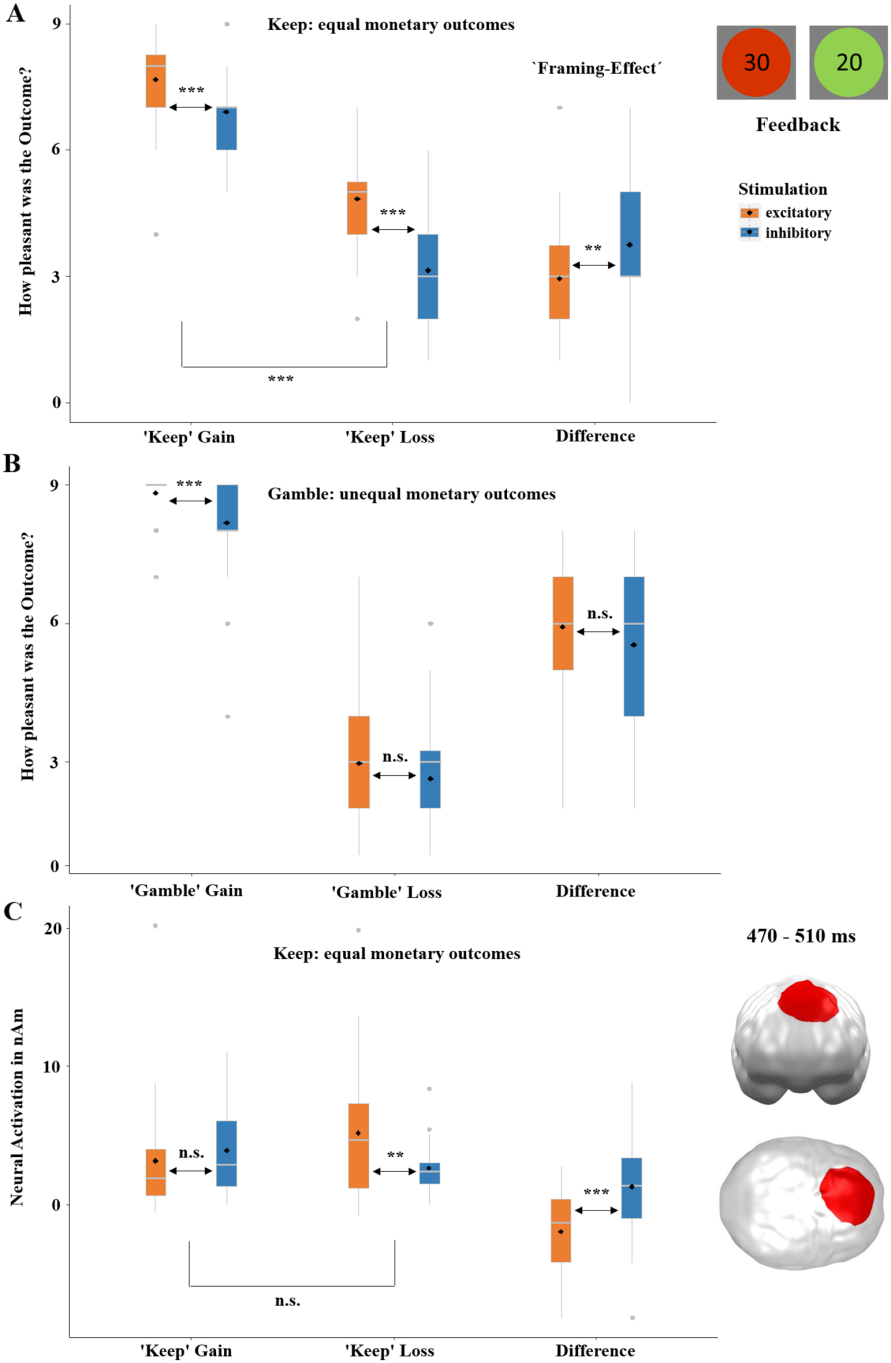
(**A**) Rated hedonic valence (pleasantness) on a SAM scale—1 (most negative) to 9 (most positive)—in the ‘keep’ condition with equal monetary outcomes. (**B**) Rated hedonic valence in the ‘gamble’ condition with unequal monetary outcomes. Excitatory stimulation led to an overall (across keep and gamble option) more positive feedback evaluation and resulted in a relatively reduced framing-effect (i.e. the difference between gain and loss ratings in the ‘keep’ option, **A**). Thus, excitatory compared to inhibitory stimulation led to an attenuated framing-effect and to more rational feedback-processing. (**C**) Significant spatio-temporal cluster in the dorsomedial prefrontal cortex featuring a significant effect of stimulation as revealed by a t-test employing the difference of gain minus loss in the relevant framing ‘keep’ option. The greater activation in response to ‘keep’ losses following excitatory compared to inhibitory stimulation suggests that excitation stimulation helped to inhibit negative feedback, leading to a reduced negative evaluation of losses in the keep option (**A**) and eventually to a more rational evaluation of feedback stimuli. Topographies of effects observed in L2-MNE were projected on standard 3D brain models for visualization. Boxplots indicate means (black dots), medians (grey lines) and lower and upper quartiles. Asterisks indicate significance levels: +  < 0.1, * < 0.05, ** < 0.01, *** < 0.001.

After the participant's choice, the feedback on win or loss was indicated by green and red circles with the amounts of the wins or losses in the middle, respectively (Fig. 3A). Finally, participants rated their subjective hedonic valence and emotional arousal in response to each outcome. Having established that neurostimulation significantly modulated the rationality of decision-making, here we aimed to determine the effects of tDCS on rational feedback-processing in particular on its modulation of the framing-effect. We addressed this question by computing a mixed effects linear regression with the predictors stimulation (excitatory, inhibitory), outcome (gain, loss) and decision (keep, gamble). A main effect of outcome (*t* = − 25.08, *p* < 0.001; Fig. 3A) confirmed that gains were rated more positive than losses. Importantly, feedback evaluations were overall (across keep and gamble decisions; Fig. 3A and B) rated more positive after excitatory than inhibitory stimulation (*t* = 2.99, *p* = 0.003). A main effect of decision (*t* = − 5.16, *p* < 0.001; keep > gamble) was mainly driven by a less negative evaluation of losses in the ‘keep’ condition after excitatory stimulation which will be further discussed below. While stimulation did not affect the factors decision and outcome alone (stimulation by decision: *t* = − 0.17, *p* = 0.867; stimulation by outcome: *t* = 1.65, *p* = 0.245), the three-way interaction was significant (stimulation by decision by outcome:* t* = 5.33, *p* < 0.001). Post-hoc repeated-measures ANOVAs that were conducted separately for the ‘keep’ and ‘gamble’ conditions revealed a significant interaction of stimulation by outcome in the ‘keep’ condition (*F*(1, 35) = 11.48, *p* = 0.001; Fig. 3A), while the respective interaction was insignificant in the 'gamble' condition (*F*(1, 35) = 1.79, *p* = 0.190; Fig. 3B) indicating that the three-way interaction was mainly driven by effects of stimulation in the 'keep' condition. To further elucidate the influence of outcome in the three-way interaction and the effect of stimulation on rational decision-making, we calculated the difference of gain-ratings minus loss-ratings after ‘keep’ and ‘gamble’ decisions and calculated t-tests comparing excitatory and inhibitory stimulation. With respect to modulations of the framing-effect, the difference of gain-ratings minus loss-ratings in the ‘keep’ condition (Fig. 3A) was of major interest since both options held the same monetary value while monetary outcomes were very different in the ‘gamble’ condition (Fig. 3B). As predicted, the ‘framing-difference’ was smaller following excitatory compared to inhibitory tDCS (*t*(35) = − 3.14, *p* = 0.003), while the corresponding t-test in the ‘gamble’ condition was insignificant (*t*(35) = 1.64, *p* = 0.111). Thus, vmPFC excitation compared to inhibition also induced a smaller framing-effect in feedback-processing, as indicated by a smaller difference between the valence ratings of the ‘keep gain’ and ‘keep loss’ condition (Fig. 3A).

**Figure 4:**
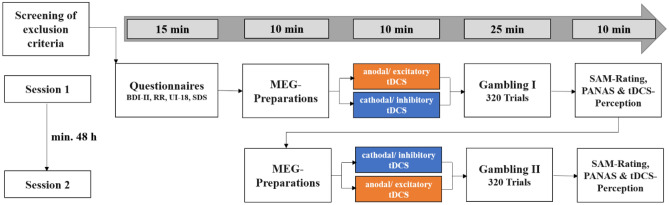
Overview over the experimental procedure. *Abbreviations:* BDI-II: Beck Depression Inventory-II. RR: Scale for Measuring Reward Responsiveness. UI-18: Intolerance of Uncertainty scale. SDS-CM: Social Desirability Scale by Crowne and Marlowe. SAM-Rating: Subjective Ratings of Hedonic Valence and Emotional Arousal. PANAS: Positive and Negative Affect Schedule. For results of the questionnaires see SM1.1.

**Figure 5:**
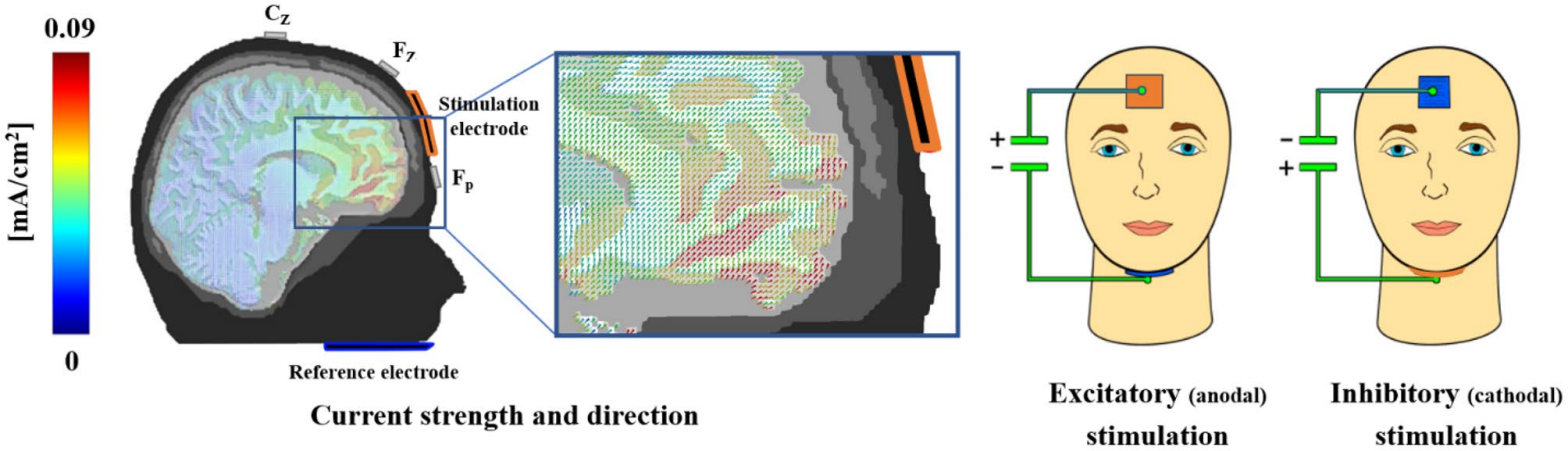
An iterative gain function algorithm aiming at maximal vmPFC-targeted stimulation revealed an electrode positioning with a small mid-frontal electrode and an expanded extracephalic chin reference. This array allowed a quasi-reference-free stimulation, providing clear differentiation of excitatory and inhibitory effects. Participants were stimulated at two different days for 10 min with 1.5 mA in an either excitatory (anodal forehead electrode) or inhibitory (cathodal forehead electrode) fashion. While the current strength is identical for anodal and cathodal stimulations, the direction of effects, as indicated by cones in the magnification, is reversed. A modeled 1.5 mA stimulation resulted in a maximum current density in the vmPFC regions of approximately 0.09 mA/cm^2^ (red colors). Actually, all sponges had the same color to prevent any inferring of the participants based on sponge color. This figure was published first in Junghoefer and coworkers, *Cerebral Cortex* (2017).

To investigate the neural responses to the feedback stimuli, we performed a 2 × 2 × 2 ANOVA employing the factors stimulation (excitatory, inhibitory), decision (keep, gamble) and outcome (gain, loss). In a repeated-measures ANOVA, this did not reveal a significant main effect of stimulation nor significant interactions with the factor stimulation. This was most likely due to the extremely strong main effect of outcome (loss >  > gain)—losses are usually perceived significantly more arousing than gains—that explained a large part of variance (see SM2.4.4). Nevertheless, we tested our specific hypothesis regarding the modulation of framing-effects via tDCS by subtracting gains from losses in the ‘keep’ option (i.e. the relevant comparison to evaluate the effect of stimulation on framing) and used the resulting difference for a t-test (excitatory versus. inhibitory). This revealed a significant cluster (*p-*cluster = 0.045) in the dorsomedial prefrontal cortex in a late time interval between 470 and 510 ms (Fig. 3C). Post-hoc tests of neural activity within this cluster revealed greater activations in response to losses after excitatory compared to after inhibitory stimulation (*t* = 2.79, *p* = 0.010) while stimulation did not modulate gain trials (*t* = − 0.79, *p* = 0.437). The increased neural responses to losses in the ‘keep’ condition following excitatory stimulation suggest facilitated inhibition of loss-aversion leading to less negative evaluations of losses (Fig. 3A) and eventually more rational evaluation of feedback stimuli.

In summary, convergent to effects in the decision-making phase, the analysis of feedback-processing implies that excitatory compared to inhibitory stimulation reduced behavioral reactivity to negative stimuli potentially driven by an enhanced inhibition of losses by mPFC regions ultimately leading to an attenuated framing-effect.

## Discussion

We investigated the contribution of the vmPFC on rational decision-making and feedback-processing by increasing or reducing its excitability via non-invasive tDCS. We found evidence for a causal role of vmPFC activity in rational decision-making on both behavioral and neural levels. Excitatory stimulation induced more adaptive decision-making compared to inhibitory tDCS, which was reflected by less susceptibility to the framing-effect, a greater likelihood to choose the option providing the higher expected value and a decreased tendency to risk larger amounts (SM2.3.3). The neural data supported this pattern, suggesting an increased ability of bilateral ventral prefrontal cortex regions to inhibit maladaptive choices (reduction of the framing-effect and less frequent choice of options with a high risk-to-lose) and thus enable more adaptive and less impulsive gambling behavior after excitatory than inhibitory stimulation. The same applied to the feedback-processing, where reduced loss-aversion (i.e. overweighting of losses^4^) after excitatory stimulation delivers a possible explanation for less susceptibility to the framing-effect in both, behavioral and neural data. Overall, our results show that non-invasive brain stimulation of the vmPFC can influence the rationality of human decision-making and feedback-processing as seen on both behavioral and neural levels.

Our results reveal that a vmPFC-targeted stimulation influences the susceptibility to the framing-effect. As seminal studies have previously shown^1,10^, participants exhibited more risk-taking behavior in the ‘loss-framed’ option, compared to the ‘gain-framed’ option. This bias was further increased by inhibitory compared to excitatory stimulation, as participants featured even more risk-taking behavior in the ‘loss-framed’ option after vmPFC-deactivation (Fig. 1B). The importance of vmPFC activation to integrate frames into decisions has been found before^23^ and is present in our neural data as well (Fig. 1C). We found greater activity in response to the ‘loss-frame’ following excitatory stimulation in the right OFC replicating a correlation of right OFC-activity and rational decision-making in the fMRI study from DeMartino and coworkers^6^. Greater OFC-activation to the ‘loss-frame’ after excitatory stimulation suggests a relative improved inhibition of a potentially irrational loss-aversion and/or risk-seeking in the loss-frame. In contrast, inhibitory stimulation could even strengthen this non-adaptive tendency and promote a more impulsive decision for the ‘gamble’ option, although the expected value may be higher in the ‘loss-framed’ option. Accordingly, a study employing delayed reward found that excitatory tDCS of the vmPFC improved the ability to wait for rewards and reduce impulsivity accordingly^22^.

Second, vmPFC excitation decreased risk-taking behavior, when the risk of losing was high (i.e. reduced risk-seeking for expected losses) and increased risk-taking, when the chance to win was high (i.e. reduced risk-aversion for expected gains), overall enhancing the expected value of decisions and the overall wins compared to inhibitory stimulation (Fig. 2B and C). This is consistent with previous findings showing that patients with vmPFC-lesions are less able to assess the probability of gains/losses and consequently win less money than healthy controls^11^. The spatiotemporal MEG cluster of stimulation by risk-to-lose overlapping the vmPFC (Fig. 2D) may illuminate the underlying neural mechanism: after inhibitory stimulation an increasing risk-to-lose came along with decreasing prefrontal activity, suggesting that vmPFC inhibition reduced the ability to suppress maladaptive choices. By contrast, excitatory tDCS facilitated this capability in participants as indicated by increasing prefrontal activity with increasing risk to lose, in turn leading to more adaptive decision-making. This result can be underlined with findings of an fMRI study, where greater activity in medial prefrontal areas occurred when risk anticipation was necessary^24^. Concluding, our results combined with previous findings strongly suggest that ventro-medial prefrontal activity is responsible for risk assessment and that this process can be modulated by non-invasive tDCS.

Another important finding is the increased tendency to gamble with increasing ‘game stakes’ after inhibitory stimulation, while risk-taking behavior did not change after vmPFC excitation (see SM2.3.3). This is consistent with the finding that vmPFC-lesioned patients typically bet more money than healthy participants^11^. Additionally, pathological gamblers show vmPFC-hypoactivation and its strength was correlated with gambling severity^25^. Combined with the finding that excitatory stimulation induced less risk-taking behavior overall and specifically in high-risk situations, these findings suggest that excitatory vmPFC-tDCS might be a promising add-on treatment option for patients suffering from gambling addiction. It should be noted, that the reported behavioral and neural effects occurred after only one rather short and mild excitatory/inhibitory tDCS (far below the thresholds considered to be safe^26^). Thus, follow-up clinical tests on add-on therapy effects have the option to use more sessions, longer durations and greater intensities of excitatory stimulation.

Now considering feedback-processing we can further elaborate the idea that the vmPFC modulates the framing-effect and, thus, is important for more rational (i.e. less biased) feedback-processing and can be modulated through tDCS. We identified that the significant interaction of stimulation by decision by outcome (Fig. 3A and B) was driven by a greater framing-effect after inhibitory stimulation. Thus, as in decision-making, more rational feedback-processing (i.e. decreased susceptibility to framing) occurred after excitatory compared to inhibitory stimulation. This can also be explained by a model that sees the vmPFC as a region tracking the (financial) value of a decision providing the basis for learning from reward and punishment. This function is implied by lesion^27^ and functional imaging^8,9^ studies, which suggest that the vmPFC is also responsible for evaluating decisions and guiding future behavior by these experiences. The relevance of the vmPFC for learning and prediction is underlined by a study on cocaine addicts^28^, in which the so called feedback-negativity, an ERP-component that is predominately modulated by vmPFC activity^29^, was examined. Cocaine addicts exhibited an impaired feedback-processing in response to losses^28^, indicating their inability to learn from losses possibly related to vmPFC-hypoactivation eventually resulting in compromised predictions.

More positive feedback ratings overall following excitatory compared to inhibitory vmPFC-tDCS also dovetail with our previous findings of a relative reduced negativity-bias of emotional face and emotional scene processing after excitatory compared to inhibitory stimulation^14–17^. Indeed, losses in the ‘keep’ option in particular were rated as less negative after excitatory compared to inhibitory stimulation, which would fit with the hypothesis that vmPFC excitation reduces loss-aversion. Convergent, losses in the ‘keep’ option also evoked stronger neural responses after excitatory compared to inhibitory stimulation (Fig. 3C). Like the strengthened neural responses to loss-frames in right anterior temporal/orbitofrontal areas (Fig. 1C) after excitatory stimulation the neural responses to ‘keep’ losses in dorsomedial prefrontal areas also suggest an enhanced inhibition of loss processing eventually leading to a less negative evaluation of ‘keep’ loss outcomes as seen in Fig. 3A. In fact, dorsolateral and dorsomedial prefrontal areas are typically active during implicit^30^ and explicit^31^ suppression of negative stimulus processing (e.g. the cognitive control of emotion).

As expected, spatiotemporal neural clusters involving stimulation and rational choice and feedback effects occurred in mid-latency or late time intervals (earliest effect starts at 220 ms). This is probably due to the nature of rational processing, as a higher-order cognitive process, that is connected downstream to sensory stimulus processing and emotionally driven decisions. ERP results confirm this pattern, where correlates of rational decision-making are typically not found earlier than the P300^32^. In comparison, clusters that are more related to impulsive decision-making have their onset already in rather early time ranges (Figs. SM3, SM4, SM5A).

Despite these new insights in vmPFC-functioning and non-invasive brain stimulation in decision-making and feedback-processing, there are limitations to consider. First, to guarantee successful blinding of participants to the stimulation conditions and to reduce inter individual variance we opted here for a within-subjects-design and first sacrificed the comparison with a sham condition (i.e. participants easily detect the difference between active and sham tDCS while both active conditions are typically undistinguishable; see SM2.1). While all conclusions regarding causal functionality and modulating capability of the vmPFC remain unaffected, it remains to be resolved whether inhibitory stimulation might solely evoke temporary vmPFC dysfunctions in healthy controls as reported in patients and/or if excitatory stimulation might improve rational decision-making even in healthy participants. To investigate this question, follow-up studies should use between-designs comparing effects of excitatory versus sham vmPFC-tDCS in healthy participants. Such designs could also be used in clinical settings to test a complementary treatment option for gambling (and other behavioral) addictions. In fact, first indications for positive effects of mPFC stimulation have been demonstrated for obsessive–compulsive disorders^33^, which is likewise associated with impaired impulse-control. The second limitation is related to the limited focality of tDCS stimulations. Since tDCS cannot exclusively target the vmPFC^34^, also frontopolar/dorsomedial prefrontal cortex regions, which are also involved in decision-making^35^, were stimulated, though with distinctly smaller intensities. Therefore, replication studies, e.g., with repetitive transcranial magnetic stimulation (rTMS) providing more focal stimulation, should be performed to further support our hypothesis that vmPFC areas are mainly responsible for the modulations of the framing-effect. Third, we cannot differentiate the contribution of loss-aversion, risk-aversion/risk-seeking, or possibly other underlying mechanisms to the altered framing-effect reported here. Further vmPFC-targeted stimulation studies directly targeting these biases should shed more light on their relative contribution to the modulation of framing-effects. Fourth, although excitatory compared to inhibitory stimulation evoked a strong and sustained increase of neural activity in the vmPFC target region as well as in the neighboring vlPFC/temporal-pole regions (Fig. SM4) it should be mentioned that the neural correlates of the modulated framing-effect were not found at the vmPFC site of stimulation. This conforms to our previous tDCS studies on emotional scene and face processing as well as fear generalization^14–17,36^, where local vmPFC-targeted stimulation effects on valence processing were verifiable neither in the fMRI nor in the MEG studies. In fact, remote neuro-stimulation network effects without local effects on the stimulated area itself have also frequently been reported by other researchers^37–39^. At last, the neural data of the feedback phase contained limited informative value, due to the extremely strong differential effects of the outcomes (loss >  > gain), which explained most variance and potentially masked relevant interaction effects. Nevertheless, we could at least partly circumvent this problem by calculating a t-test, that was justified by our hypotheses and by the behavioral effects.

## Conclusion

Our results not only support the claim that the vmPFC plays a causal role for rational decision-making and feedback-processing, but they also indicate that rationality can be modulated by its non-invasive stimulation. Improved decision-making after excitatory compared to after inhibitory vmPFC-tDCS as reflected in maximized wins and decreased susceptibility to the framing-effect is a compelling illustration of this influence. The same is true for the feedback phase, where less loss-biased stimulus processing might guide more adaptive behavior in the future. These higher-order cognitive processes were reflected by neural activity in prefrontal clusters at mid-latency to late time intervals. Our results suggest that the reduced framing effect and the resulting more rational decision making are primarily due to relatively attenuated negative affective biases (i.e. loss-aversion and risk-aversion). The finding that vmPFC excitation compared to inhibition can induce more rational decision-making and feedback-processing raises the hope for clinical applications of vmPFC-tDCS in addiction disorders such as pathological gambling.

## Methods

### Participants

We included 37 (17 women) right-handed volunteers, aged 19–29 years (*M* = 23.42, *SD* = 2.70) meeting the inclusion criteria (see SM1.1). Participants were pseudo-randomly assigned to experimental groups that were matched regarding demographic and psychometric features. The study was ethically approved by the ethics committee of the medical school at the University of Münster. All research has been performed in accordance with the Declaration of Helsinki.

Participants were told a cover story to ensure authentic gambling behavior. It was stated that they could win an amount between 0 and 36€ in addition to the fixed allowance of 30€. Following the study, participants were elucidated regarding the cover story and everybody received the full amount of 66€.

### Specifications on the gambling task

Each trial began with a fixation cross after which the initial amount of money was presented, for which the participants could successively gamble with a specified risk (game stake of 25ct, 50ct, 75ct or 100ct). Second, the ‘choice stimulus’ appeared on which basis the participants had to decide whether they would choose a safe (‘keep’) or a risky (‘gamble’) option. The framing-effect is relevant in the ‘keep’ option: If the game stake was for instance 50ct (Fig. 1A), the green gain-frame informed about a safe win of 20ct (i.e. equivalent to a safe loss of 30ct) while the red loss-frame predicted a safe loss of 30ct (i.e. equivalent to a safe win of 20ct). According to this scheme the gain-frames informed about safe wins of 10ct, 30ct, or 40ct and the loss-frames of safe losses of 15ct, 45ct, or 60ct, if the initial amounts were 25ct, 75ct or 100ct, respectively.

### Experimental procedure

In a within-subjects-design each participant received excitatory/anodal and inhibitory/cathodal stimulation over the course of two sessions with a minimum-interval of 48 h between the sessions (Fig. 4). The allocation of stimulation order (excitatory or inhibitory stimulation first) was randomized across participants. In the beginning of the first session, participants gave written informed consent, filled questionnaires, comprising the Beck Depression Inventory^40^, the Reward Responsiveness scale^41^, the Intolerance of Uncertainty scale^42^, and the Social Desirability Scale^43^. After tDC stimulation, participants executed the gambling task in the MEG, where event-related fields (ERFs) in response to the choice- and feedback stimuli were measured. At the end of each session, participants rated the feedback regarding subjective hedonic valence and emotional arousal on a self-assessment manikin (SAM) rating scale^44^, rated their state mood on the Positive and Negative Affect Schedule (PANAS^45^) and rated their perceived stimulation pleasantness and stimulation intensity on an in-house questionnaire. In the second session, the same procedure was used with the opposite stimulation polarity. Finally, subjects were elucidated about the cover story. The duration of both sessions combined was approximately 200 min (Fig. 4).

### tDCS

Transcranial direct current stimulation (tDCS) is a widespread and effective method to modulate brain activity from outside the skull. An anodal or excitatory stimulation depolarizes the membrane potential of neurons, what increases their excitability depending on the strength of the applied electric field. In contrast, cathodal or inhibitory stimulation hyperpolarizes the neuron membrane, reducing the likelihood of action potentials^26^. An important advantage of tDCS is the low rate of side effects (e.g. headache, nausea and insomnia) and, with particular relevance for ventral prefrontal target regions, the absence of unwanted co-stimulation of facial and ocular muscles and nerves. Changes of cortical excitability can last up to one hour after a single stimulation^46^. We implemented the tDCS-montage as used in our previous studies to non-invasively stimulate the vmPFC^14–17,36^. The active electrode was placed on the forehead (3 × 3 cm) and the extracephalic reference under the chin (5 × 5 cm). Electrodes were plugged into sponges that were soaked with a sodium-chloride solution to ensure electric conductivity. For excitatory/anodal or inhibitory/cathodal stimulation, the forehead electrode was used as anode or as cathode respectively. This electrode-montage results in maximal stimulation of the vmPFC and minimal stimulation of adjacent brain regions, as revealed by finite-element based forward modelling of tDCS currents^47^. Using a DC Stimulator Plus (NeuroConn GmbH), we applied a maximum current of 1.5 mA for 10 min with both stimulation polarities.

### Recording and preprocessing of MEG

Event-related fields were measured using a 275 whole-head sensor system (CTF Systems, first-order axial gradiometers) with a sampling rate of 600 Hz across a frequency range from 0 to 150 Hz (anti-aliasing hardware filtering). The continuous data were down-sampled to 300 Hz and filtered with a 0.1 high-pass-filter and 48 Hz low-pass-filter. We extracted epochs from 200 ms before and 600 ms after stimulus onset and employed the interval of − 150 ms to 0 ms for baseline adjustment. To identify and reject artifacts the method suggested by Junghöfer and colleagues was used^48^. With this method, individual and global artifacts were discovered. In case noisy channels were identified, their signal was estimated by spherical-spline-interpolation based on the weighted signal of all remaining sensors. A minimum threshold of 0.01 for the estimated Goodness of Interpolation was applied and trials exceeding this value were rejected. If more than 30% of the trials were discarded in any session, the respective participant was excluded from further analysis (three participants). Trials within each experimental condition were averaged for each participant and session and the underlying neural sources of the measured ERFs were estimated by applying L2-Minimum-Norm-Estimates^49^ (L2-MNE). The preprocessing and analysis of the MEG data was performed with the MATLAB (2019b)-based EMEGS software^50^ (version 3.1). For details see SM1.2.

### Data-analysis

We used mixed effects models for the behavioral data because of their robustness in repeated-measures compared to conventional models^51^. Detected effects were then resolved with traditional post-hoc tests.

Aiming to test which factors influence rational decision-making (choice to ‘keep’ or ‘gamble’) we calculated a mixed effects logistic regression with the predictors stimulation (excitatory, inhibitory), risk-to-lose (20, 40, 60, 80%) and frame (gain-frame, loss-frame). Since we were mainly interested in the interaction effects of stimulation by frame and stimulation by risk-to-lose we modeled random effects for these interactions. The overall model was significant (*χ*^2^(16) = 13,297.00, *p* < 0.001) and within this model all main effects got significant: stimulation (*z* = − 9.69, *p* < 0.001), risk-to-lose (*z* = − 9.60, *p* < 0.001) and frame (*z* = 2.64 , *p* = 0.008). The interaction of stimulation by frame was insignificant (*z* = 0.39, *p* = 0.695), but since we had specific hypothesis regarding this effect, we calculated a separate logistic regression with only these predictors. As expected, the interaction of stimulation by frame was significant in this model (*z* = 2.19, *p* = 0.029). However, the interaction of stimulation by risk-to-lose got highly significant in the overall model (*z* = 6.60, *p* < 0.001).

The neural analyses in the decision-making phase (stimulation by frame by decision and stimulation by risk-to-lose) were performed separately from each other to ensure a sufficient number of trials per condition for a reasonable signal-to-noise-ratio in the source estimation. To correct for multiple comparisons we applied a non-parametric approach proposed by Maris and Oostenveld^52^. For details see SM1.4.For the analysis of the perceived hedonic valence (SAM-rating^44^), 9-point Likert scale) of the feedback, we computed a mixed effects linear regression with the predictors stimulation (excitatory, inhibitory), outcome (gain, loss) and decision (keep, gamble). Since we were mainly interested in the interaction effects of stimulation by decision, we modeled random effects for this interaction.

The overall model was significant (*χ*^2^(9) = 526.44, *p* < 0.001) and revealed significant main effects of stimulation (*t* = − 2.99, *p* = 0.003), outcome (*t* = − 25.08, *p* < 0.001) and decision (*t* = − 5.16, *p* < 0.001). While the two-way-interactions of stimulation by decision (*t* = − 0.17, *p* = 0.867) and stimulation by outcome (*t* = 1.65, *p* = 0.245) were insignificant, the three-way-interaction got highly significant (outcome by decision by stimulation:* t* = 5.33, *p* < 0.001).

## Supplementary Information

Supplementary Information 1.

Supplementary Information 2.

Supplementary Information 3.

Supplementary Information 4.

Supplementary Information 5.

Supplementary Information 6.

Supplementary Information 7.

Supplementary Information 8.

Supplementary Information 9.

Supplementary Information 10.

Supplementary Information 11.

Supplementary Information 12.

## Data Availability

The datasets generated and analysed during the current study are available in the repository: https://gin.g-node.org/NeuroIBB/GametDCSFrame.
