# Supplementary figures and images for: Noninvasive stimulation of the ventromedial prefrontal cortex modulates rationality of human decision-making
Source: Sci Rep. 2022 Nov 23;12:20213. doi: 10.1038/s41598-022-24526-6 (PMC9684418; doi:10.1038/s41598-022-24526-6)

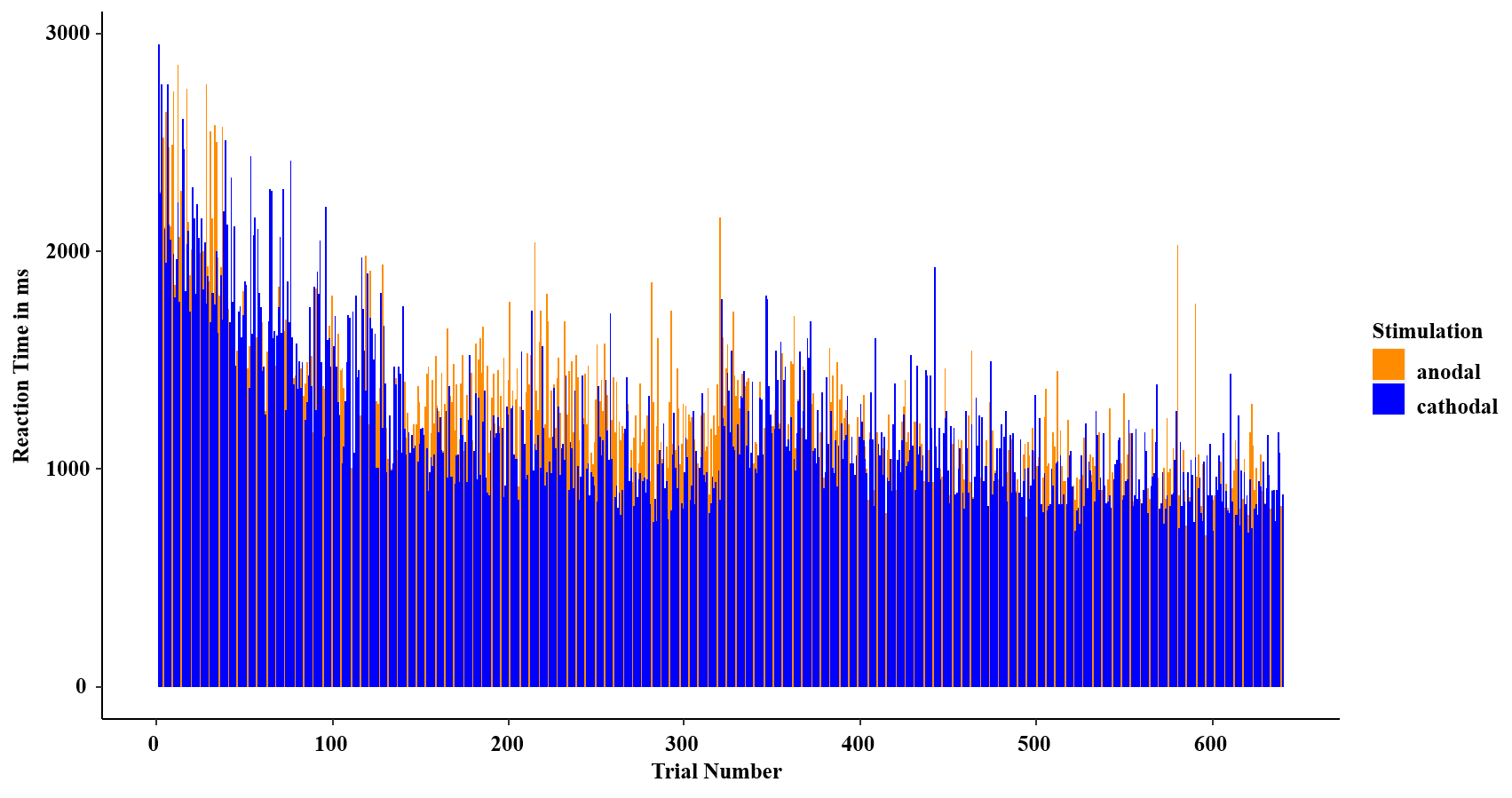

Supplement: Supplementary file 1 — Supplementary Information 1. [file 41598_2022_24526_MOESM1_ESM.tif]

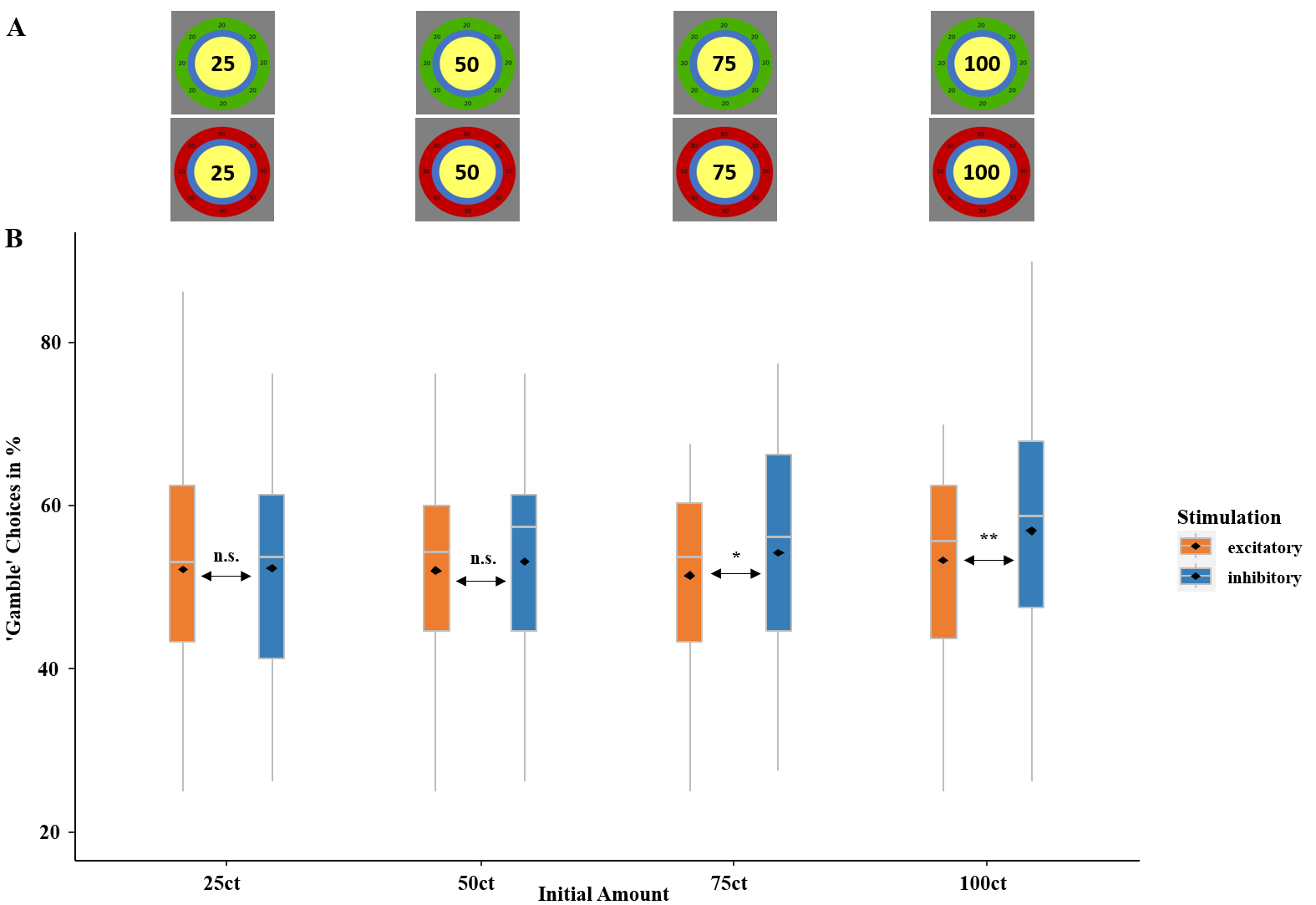

Supplement: Supplementary file 2 — Supplementary Information 2. [file 41598_2022_24526_MOESM2_ESM.tif]

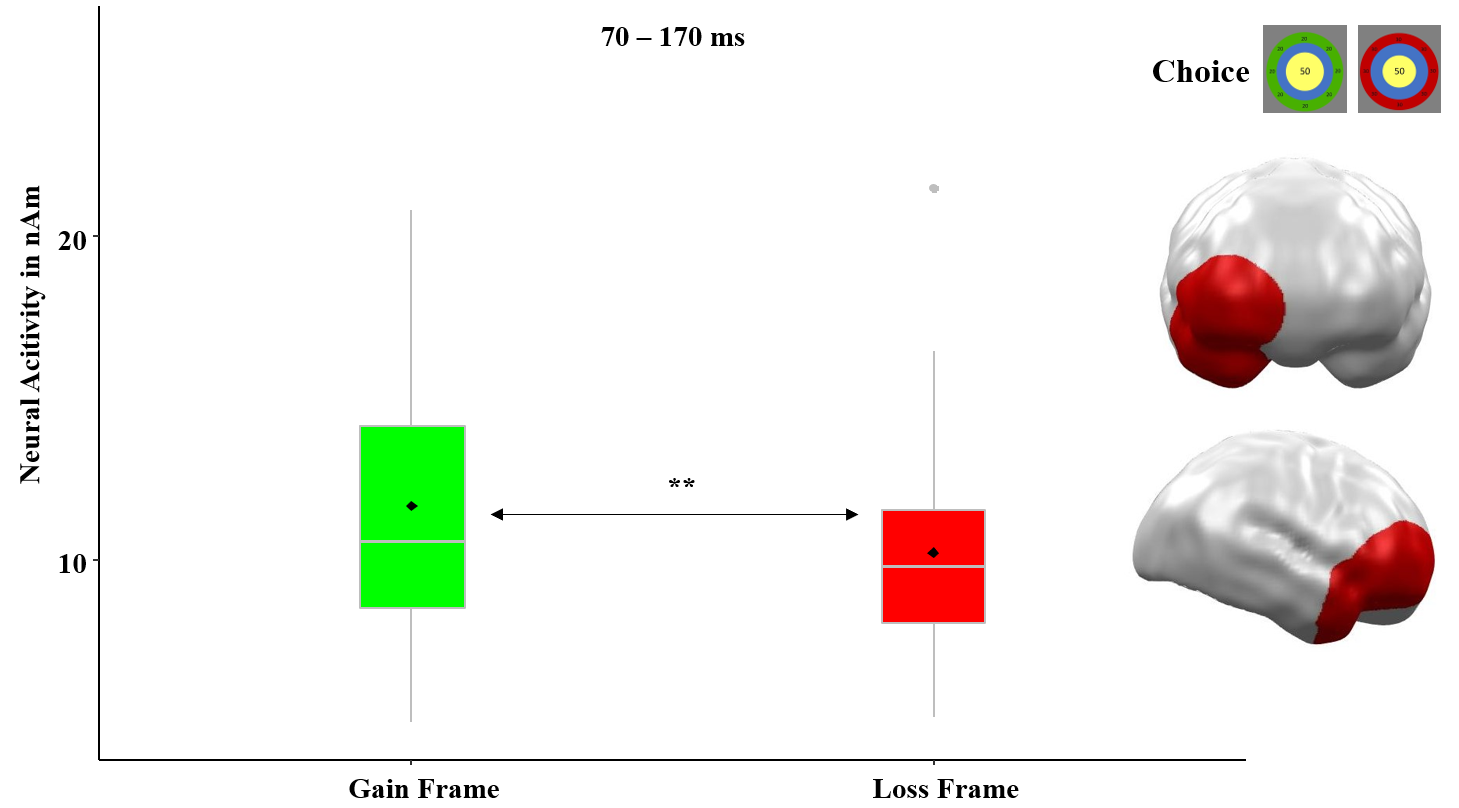

Supplement: Supplementary file 3 — Supplementary Information 3. [file 41598_2022_24526_MOESM3_ESM.tif]

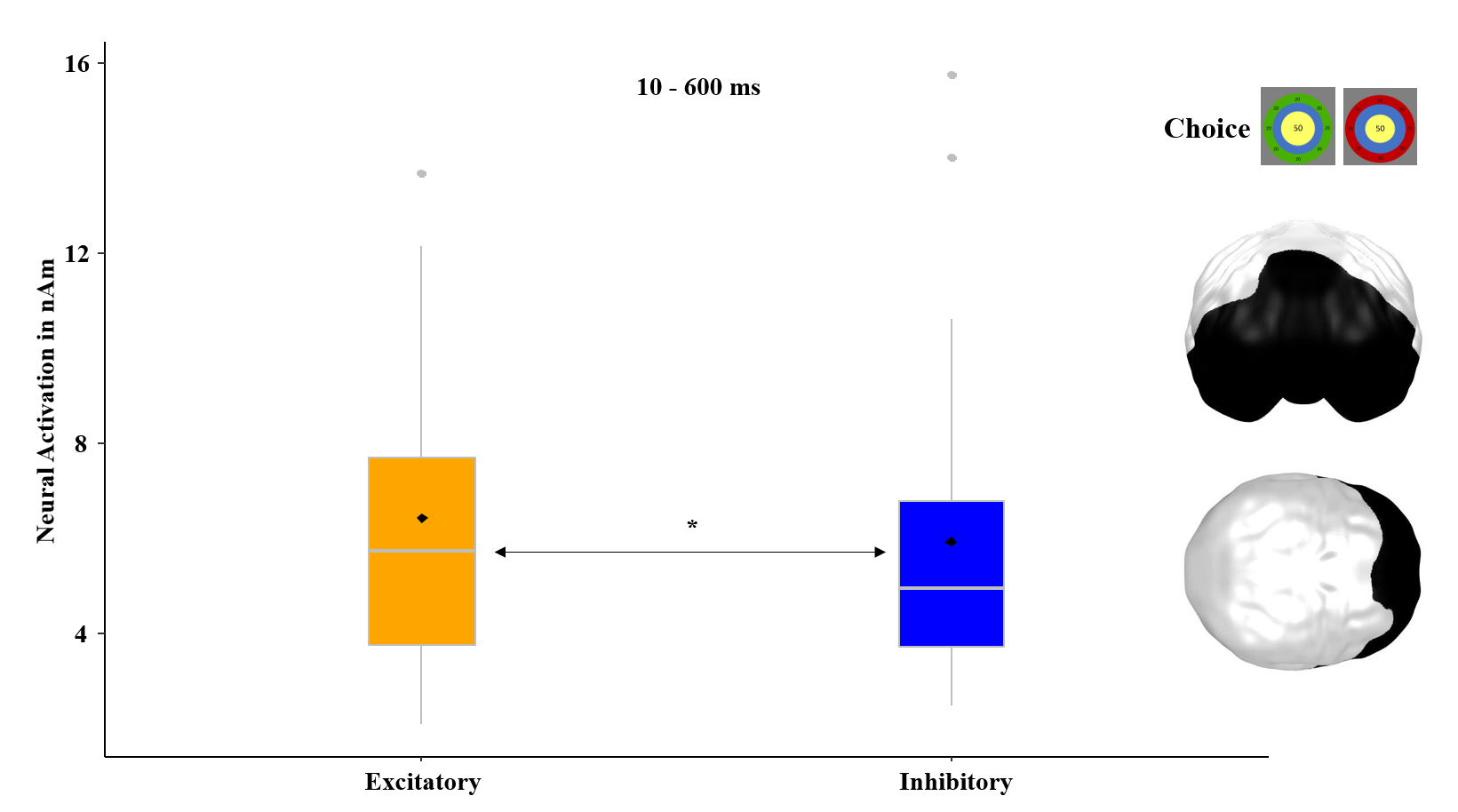

Supplement: Supplementary file 4 — Supplementary Information 4. [file 41598_2022_24526_MOESM4_ESM.tif]

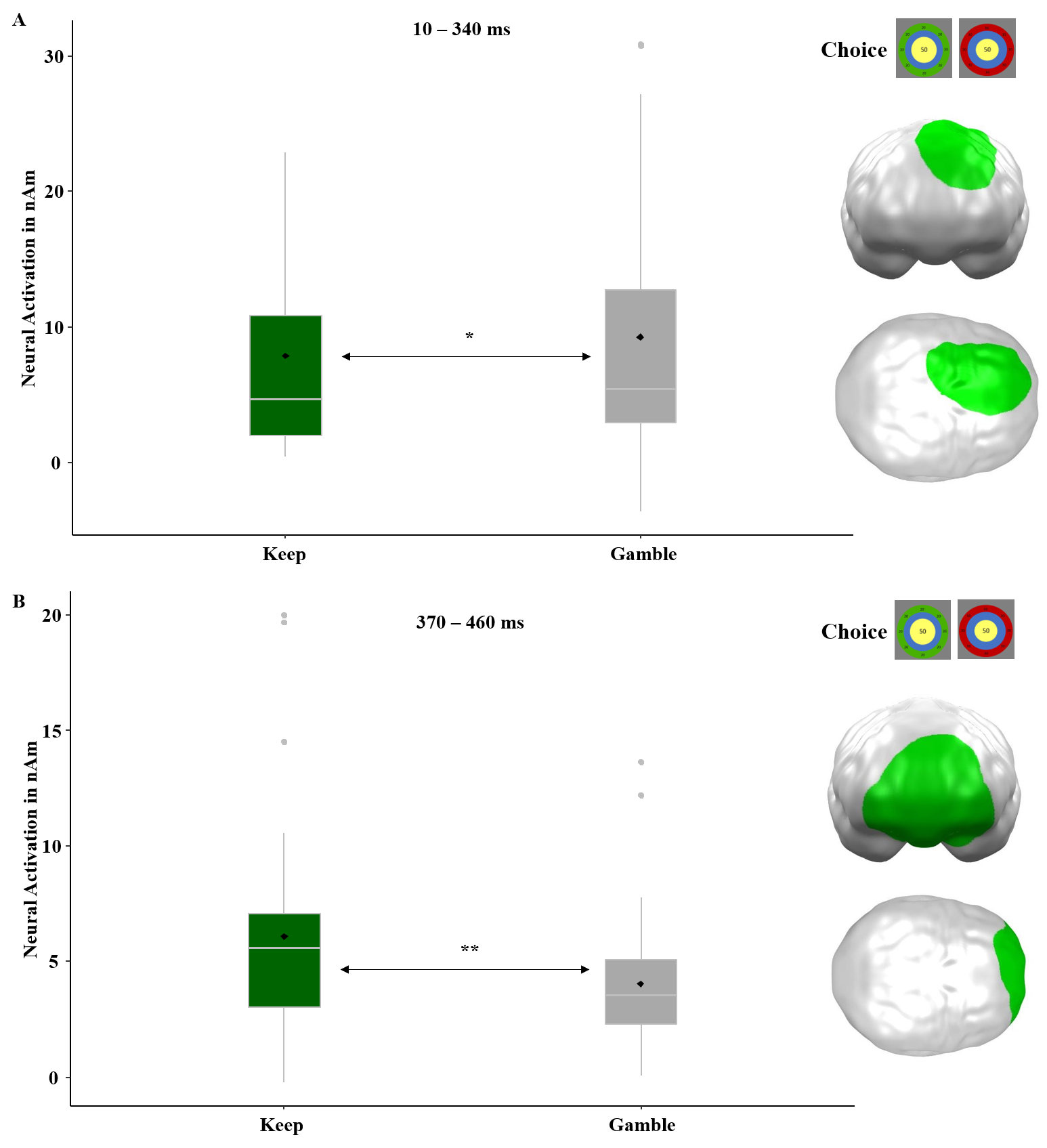

Supplement: Supplementary file 5 — Supplementary Information 5. [file 41598_2022_24526_MOESM5_ESM.tif]

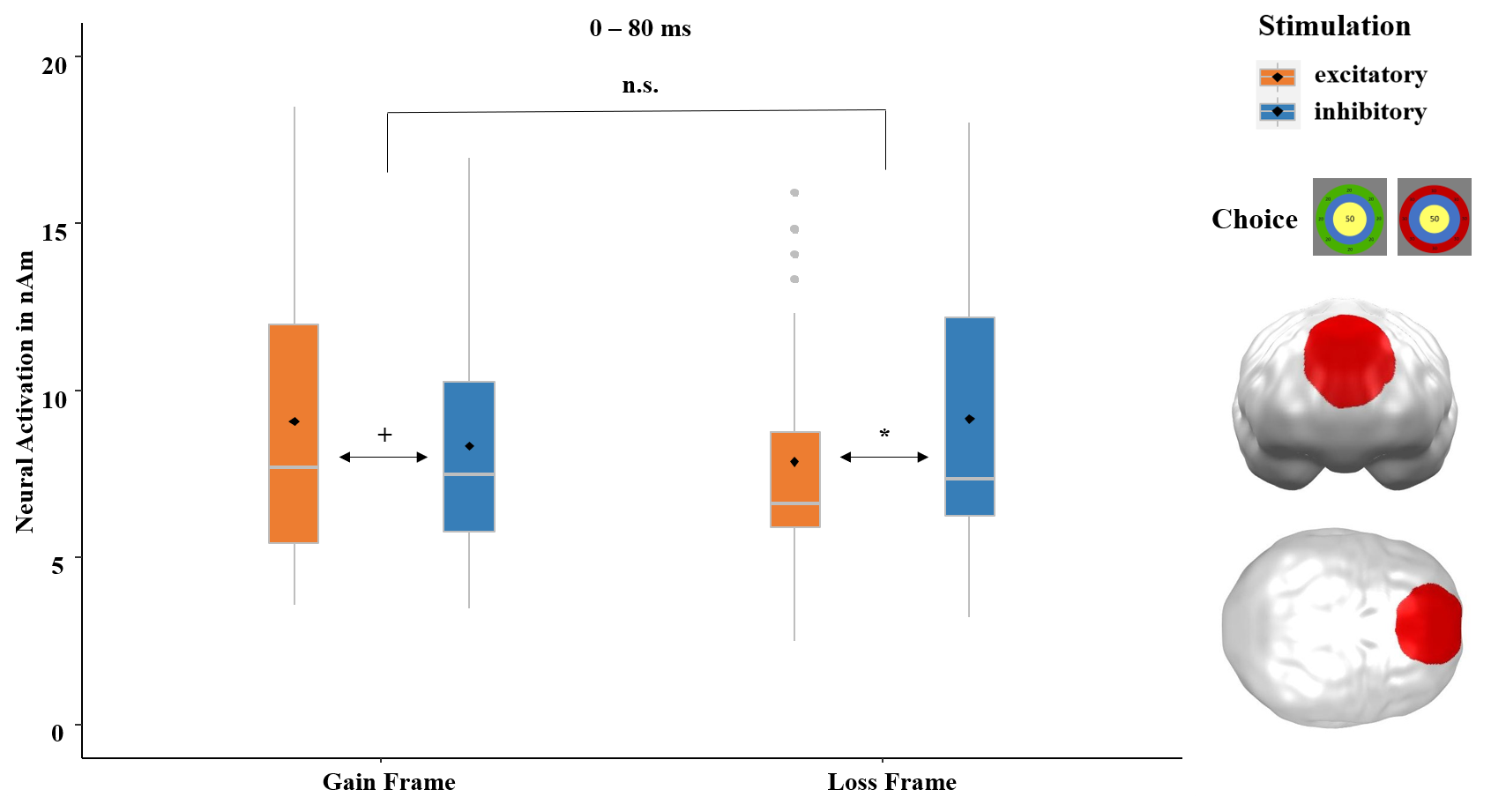

Supplement: Supplementary file 6 — Supplementary Information 6. [file 41598_2022_24526_MOESM6_ESM.tif]

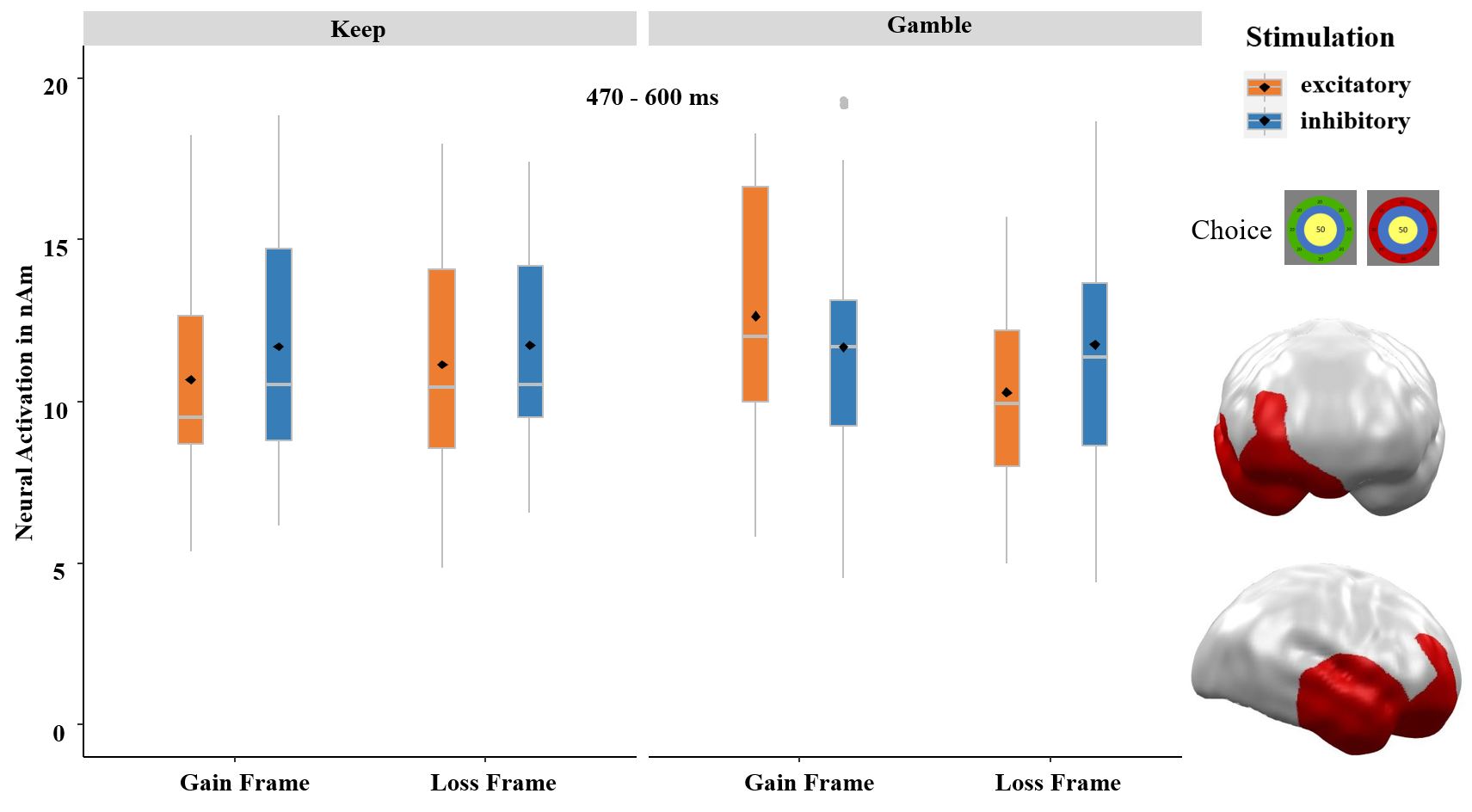

Supplement: Supplementary file 7 — Supplementary Information 7. [file 41598_2022_24526_MOESM7_ESM.tif]

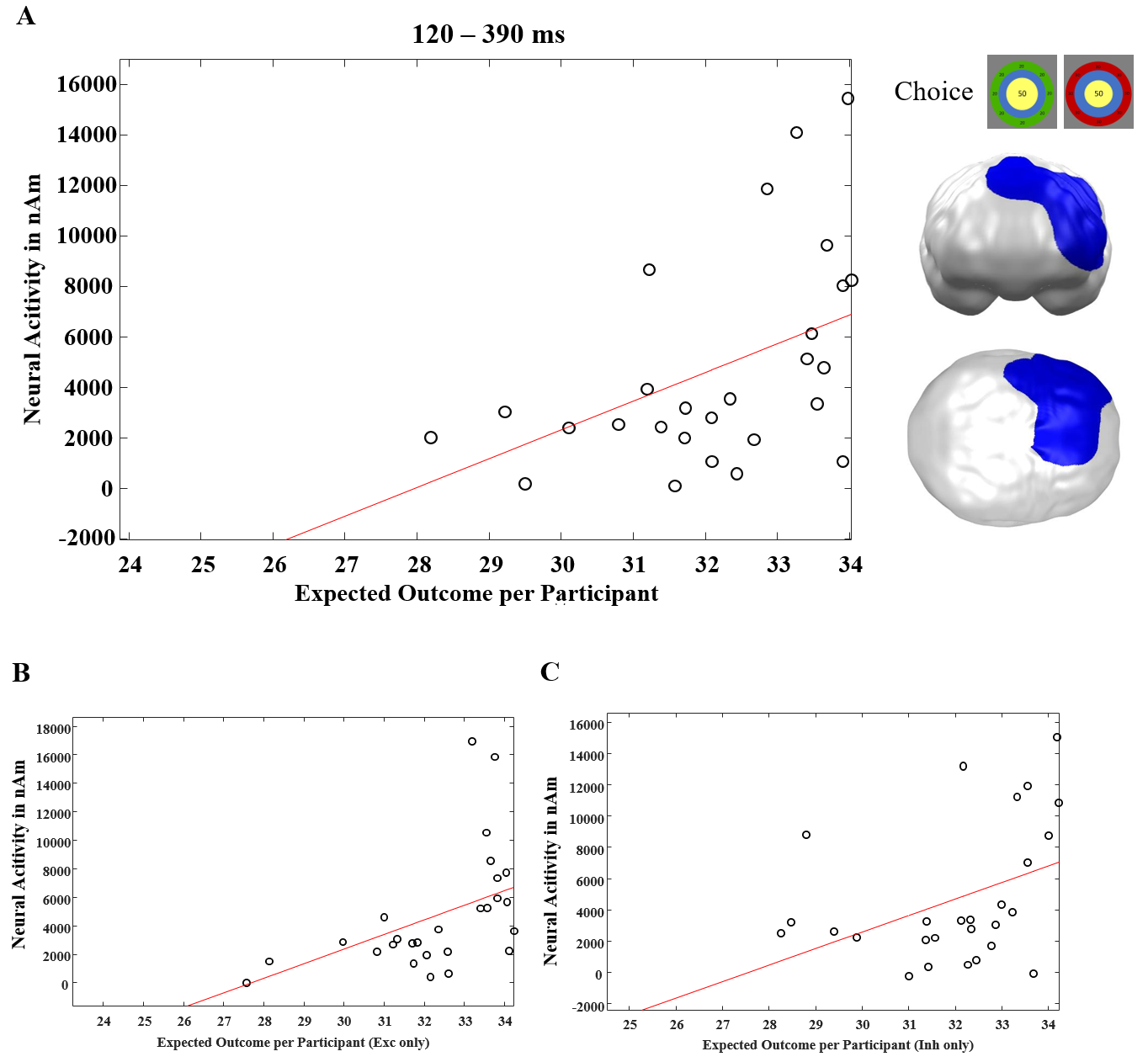

Supplement: Supplementary file 8 — Supplementary Information 8. [file 41598_2022_24526_MOESM8_ESM.tif]

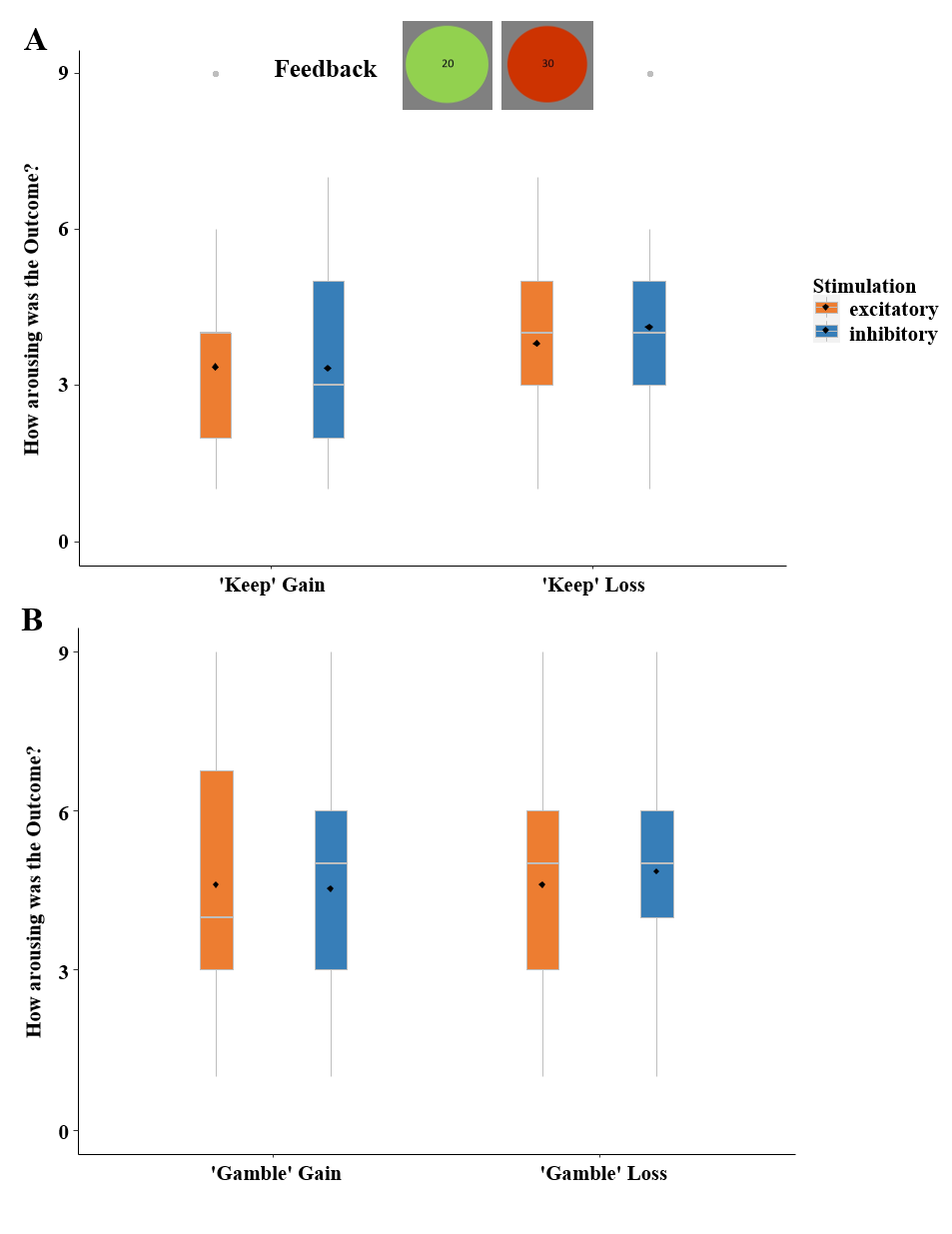

Supplement: Supplementary file 9 — Supplementary Information 9. [file 41598_2022_24526_MOESM9_ESM.tif]

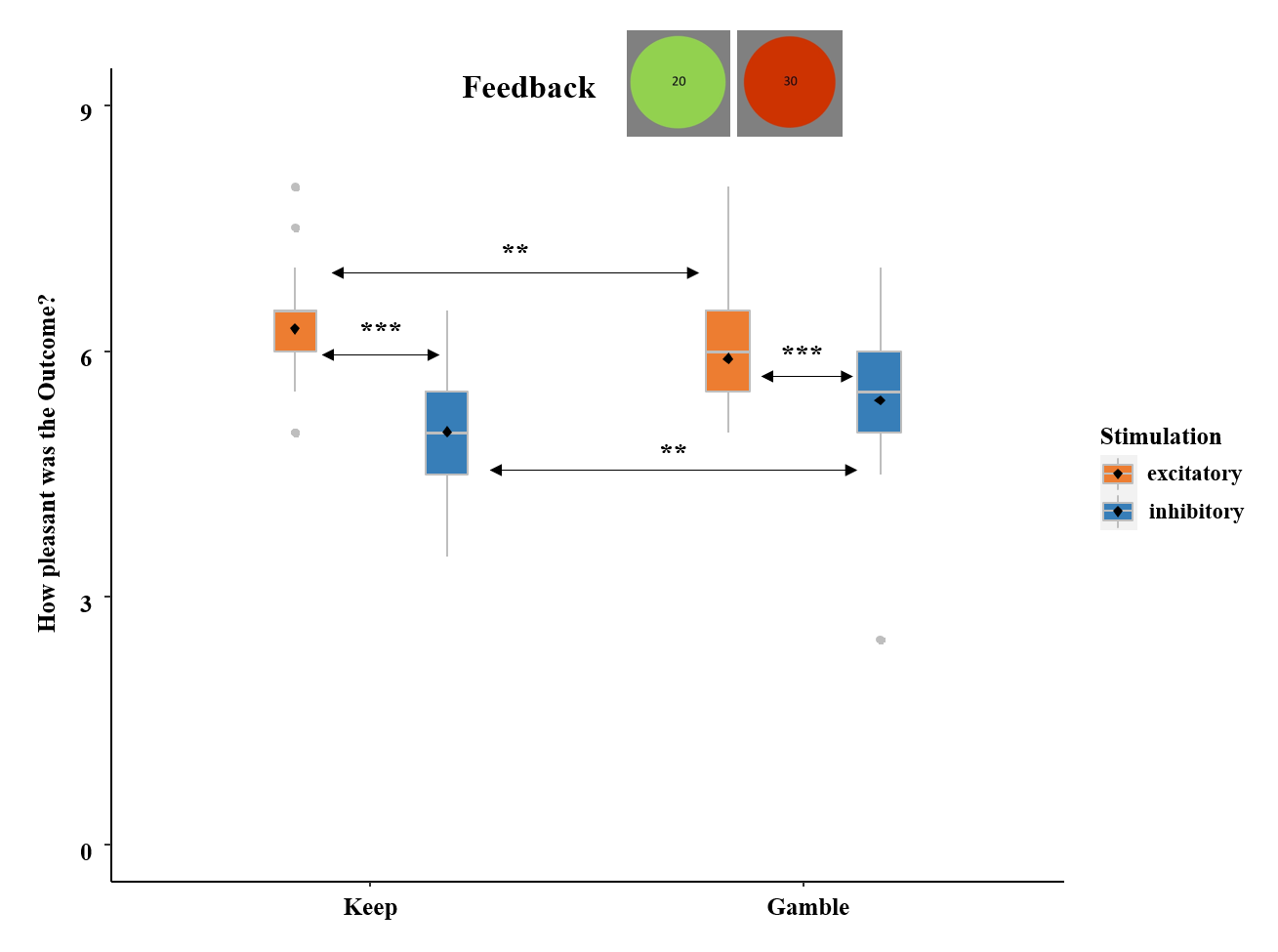

Supplement: Supplementary file 10 — Supplementary Information 10. [file 41598_2022_24526_MOESM10_ESM.tif]

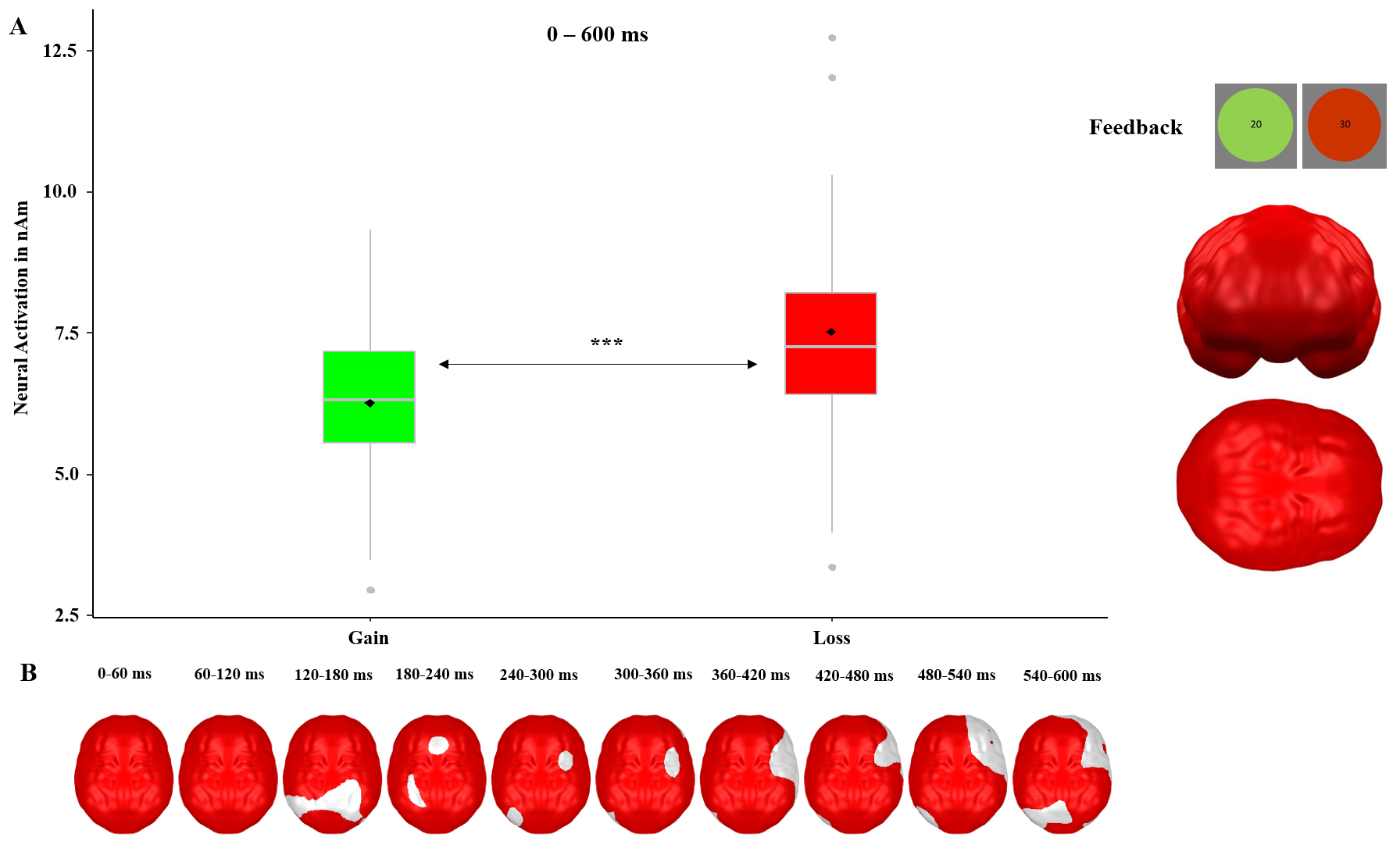

Supplement: Supplementary file 11 — Supplementary Information 11. [file 41598_2022_24526_MOESM11_ESM.tif]
